# Perceived ethnic discrimination in relation to smoking and alcohol consumption in ethnic minority groups in The Netherlands: the HELIUS study

**DOI:** 10.1007/s00038-017-0977-2

**Published:** 2017-05-16

**Authors:** Marlies J. Visser, Umar Z. Ikram, Eske M. Derks, Marieke B. Snijder, Anton E. Kunst

**Affiliations:** 1 Department of Public Health, Academic Medical Center, University of Amsterdam, P.O. Box 22660, 1100 DD Amsterdam, The Netherlands; 2 Department of Psychiatry, Academic Medical Center, University of Amsterdam, Amsterdam, The Netherlands; 3 QIMR Berghofer, Translational Neurogenomics Group, Brisbane, Australia

**Keywords:** Perceived ethnic discrimination, Smoking, Alcohol consumption, Ethnic differences, HELIUS study

## Abstract

**Objectives:**

We examined the associations of perceived ethnic discrimination (PED) with smoking and alcohol consumption in ethnic minority groups residing in a middle-sized European city.

**Methods:**

Data were derived from the HELIUS study in Amsterdam, The Netherlands. We included 23,126 participants aged 18–70 years of Dutch, South-Asian Surinamese, African Surinamese, Ghanaian, Turkish, and Moroccan origin. We collected self-reported data on PED, current smoking, heavy smoking, nicotine dependence, current drinking, excessive drinking, and alcohol dependence. Logistic regression was used.

**Results:**

In general, we observed positive associations in participants of African Surinamese and Ghanaian origin, but no associations in those of South-Asian Surinamese, Turkish, or Moroccan origin. In African Surinamese, the associations were positive for current smoking, nicotine, and alcohol dependence (odds ratios of 1.16; 95% confidence interval: 1.06–1.27, 1.34; 1.15–1.57 and 1.40; 1.20–1.64, respectively). In Ghanaians, positive association was observed for current drinking (1.21; 1.08–1.36).

**Conclusions:**

The associations of PED with smoking and alcohol consumption considerably varied by ethnicity and outcome measure. This suggests that ethnic minority groups in Europe might use different behavioural strategies to cope with PED.

**Electronic supplementary material:**

The online version of this article (doi:10.1007/s00038-017-0977-2) contains supplementary material, which is available to authorized users.

## Introduction

Perceived ethnic discrimination (PED) refers to the experience of unfair treatment because of ethnic background (Gee et al. 2009). PED is considered an important chronic psychosocial stressor for ethnic minority groups, and is associated with poorer mental and physical health (Williams et al. 2008; Ikram et al. 2015).

Discriminatory experiences might generate stress, which can lead one to adopt adverse coping responses such as unhealthy behaviours (Williams and Mohammed 2009). In fact, it has been suggested that unhealthy behaviours such as smoking and alcohol consumption might serve as a coping strategy to deal with discrimination (Williams et al. 2008). The previous studies show that perceived racial/ethnic discrimination is related to smoking (Bennett et al. 2005; Landrine et al. 2006; Borrell et al. 2007; Chae et al. 2008; Borrell et al. 2010) and alcohol consumption (Terrell et al. 2006; Borrell et al. 2007; Gee et al. 2007; Borrell et al. 2010; Gilbert and Zemore 2016). For example, Landrine et al. (2006) found that Latinos, Blacks, and Asians who reported ethnic discrimination showed higher smoking rates. Another study showed higher odds of heavy drinking in African Americans and Hispanics who reported perceived racial/ethnic discrimination (Borrell et al. 2010).

However, most studies on smoking and alcohol consumption in relation to ethnic discrimination were conducted in the United States (US), focusing mainly on African Americans. There are relatively few European studies on this topic. As European societies are becoming increasingly ethnically diverse and official reports indicate that ethnic minority groups experience discrimination in different settings (e.g., jobs and housing) (Zick et al. 2008), this suggests that discrimination could have public health relevance in Europe, as well. Important to note, findings from the US cannot be readily applicable to the European context, given the differences between European-based ethnic minority groups and those living in the US, in terms of migration history and country of origin, among other factors.

This study aimed at examining the association of PED with smoking and alcohol consumption among several ethnic groups based in Amsterdam, The Netherlands. We investigated the association of PED with current smoking, heavy smoking, and nicotine dependence, as well as with current drinking, excessive drinking, and alcohol dependence. This allows assessing the relationship between PED and different substance use outcomes. This study may help deepen our understanding on the public health relevance of PED within the European context.

## Methods

### Study population

We used baseline data from the healthy life in an urban setting (HELIUS) study, an on-going multi-ethnic cohort study conducted in Amsterdam, The Netherlands. The study protocol has been described elsewhere (Stronks et al. 2013). Briefly, the HELIUS study randomly sampled participants aged 18–70 years stratified by ethnicity, through the municipality registry of Amsterdam, which includes information on the country of birth of the participants and their parents. Subjects were sent an invitation letter (and a reminder after 2 weeks) by mail. We were able to contact 55% of those invited (55% among Dutch, 62% among Surinamese, 46% among Turks, 48% among Moroccans, and 57% among Ghanaians), either by response card or after a home visit by an ethnically matched interviewer (speaking both Dutch and the native language). Of those contacted, about 50% agreed to participate (participation rate; 60% among Dutch, 51% among Surinamese, 41% among Turks, 43% among Moroccans, and 61% among Ghanaians). Therefore, the overall response rate was 28% with some variations across ethnic groups (33% among Dutch, 31% among Surinamese, 22% among Turks, 21% among Moroccans, and 35% among Ghanaians). After a positive response, participants received a confirmation letter of the appointment for the physical examination, including a digital or paper version of the questionnaire (depending on the preference of the subject). The questionnaire was additionally translated into English for Ghanaian participants and into Turkish for Turkish participants. Participants who were unable to complete the questionnaire themselves were offered assistance from a trained ethnically matched interviewer speaking the preferred language. Non-response analyses showed no differences between participants and non-participants in socio-economic characteristics (Snijder et al. submitted). The HELIUS study has been approved by the Institutional Review Board of the Academic Medical Center, University of Amsterdam.

Baseline data were collected from January 2011 to December 2015. From the total sample of participants who filled in the questionnaire (*N* = 23,942), we excluded participants of Javanese Surinamese (*N* = 250) or unknown Surinamese origin (*N* = 286) because of the small sample sizes. We also excluded participants with an unknown or other ethnic origin (*N* = 50). Furthermore, participants with missing data on PED were excluded (*N* = 230). This resulted in a sample of 23,126 participants: 4626 Dutch, 3343 South-Asian Surinamese, 4414 African Surinamese, 2441 Ghanaian, 4012 Turkish, and 4290 Moroccan. It should be noted that participants with missing data on other covariates and the outcome measures were excluded in the corresponding analysis only.

### Variables

#### Ethnicity

Participant’s ethnicity was defined according to the country of birth of the participant as well as that of the parents, which is currently the most widely accepted and most valid assessment of ethnicity in The Netherlands (Stronks et al. 2013). Participants were considered of Dutch origin if the participant and both parents were born in The Netherlands. Participants were considered of non-Dutch ethnic origin if either they themselves were born outside The Netherlands and at least one of their parents (first-generation), or they themselves were born in The Netherlands, but at least one of their parents was born outside The Netherlands (second-generation). Participants of Surinamese origin were further subdivided (through self-reported ethnicity) into subgroups: African, South-Asian, Javanese, or other/unknown Surinamese origin.

#### Perceived ethnic discrimination

PED was conceptualized as the experiences of interpersonal discrimination based on ethnic background in daily life. We measured PED using the everyday discrimination scale (EDS) (Forman et al. 1997). EDS measures the frequency of discriminatory experiences in daily life and is based on the qualitative work done among African American women in the US and African Surinamese women in The Netherlands (Essed 1991). EDS uses nine items (e.g., ‘people treat you with less respect’; ‘people treat you less kindly’) with response scale varying from 1 (never) to 5 (very often). We modified the EDS, so that participants were specifically asked about experiences of discrimination based on their ethnic background. Mean scores of the nine items were calculated. PED was considered missing if more than one item was missing. If only one item was missing, we used the mean score of the other items to substitute the missing item.

#### Smoking

Three measures were used to distinguish between different smoking behaviours. Current smoking was assessed as smoking one or more cigarettes currently on daily basis (y/n). Heavy smoking was defined as smoking ≥10 cigarettes daily (y/n). Often, studies use a cutoff of ≥20 cigarettes for heavy smoking (Neumann et al. 2013), which may represent nicotine dependence. To clearly distinguish between the smoking behaviours and since we regarded heavy smoking as intermediary measure between current smoking and nicotine dependence, we decided to use the cutoff ≥10. Nicotine dependence was determined by the Fagerström scale (Heatherton et al. 1991) consisting of six questions (e.g., ‘do you find it hard not to smoke in places where it is not allowed’). The sum score varied from 0 to 10, with a cutoff of ≥4 considered nicotine dependence (y/n). If one of the items was missing, the Fagerström sum score was calculated with a score of 0 for the missing item. If more than one item was missing, the Fagerström sum score was coded as missing. For non- or ex-smokers, the sum score is 0.

#### Alcohol consumption

For alcohol consumption, we also used three measures. Current drinking was determined by asking whether one has used alcohol in the preceding 12 months (y/n). Excessive drinking was determined by asking how often one drinks alcoholic beverages in combination with how many glasses one drinks on a typical day of drinking. Drinking of alcoholic beverages ‘more than four times a week’ combined with ‘at least 3–4 glasses per day’ was considered excessive daily drinking (y/n). The cutoffs for excessive drinking were based on The Netherlands Mental Health Survey and Incidence Study-2 (Graaf et al. 2010). This measure does not include binge drinking behaviours as studies have indicated that social factors (e.g., drinking peers), rather than psychosocial stressors, are main determinants of binge drinking (Courtney and Polich 2009). Alcohol dependence was determined by the Alcohol Use Disorder Identification Test (AUDIT) (Babor et al. 2001), which consisting of ten questions (e.g., ‘how often during the last year have you failed to do what was normally expected from you because of drinking’). The sum score varied from 0 to 40, with a cutoff of ≥8 for determining alcohol dependence (Babor et al. 2001). If only one item was missing, the mean score of the other nine items was used to substitute the missing item. If more than one item was missing, the AUDIT was not calculated and considered missing.

#### Covariates

Other covariates included age, sex, educational level, employment status, marital status, and other psychosocial stressors. Educational level was categorized into: no education or elementary education; lower vocational or lower secondary education; intermediate vocational and intermediate/higher secondary education; and higher vocational education or university. Employment status was categorized into three categories: not in the labour force (e.g., incapacitated for work and retirement), unemployed, and employed. Other psychosocial stressors were determined with two items: any negative life events (e.g., ‘you were seriously ill or injured’) in the last 12 months (y/n) (Rosengren et al. 2004) and feeling distressed at home (never, some periods, several periods, and constantly) (Rosengren et al. 2004).

### Statistical analysis

We calculated the crude prevalence of the different smoking and alcohol measures. Since the prevalence of heavy smoking and nicotine dependence as well as excessive drinking and alcohol dependence was low in the total sample, we presented the prevalence within the sample of current smokers or drinkers, respectively. To assess ethnic differences in smoking and alcohol consumption, we calculated the age- and sex-adjusted odds ratios for smoking and alcohol consumption using logistic regression.

We assessed the association of PED with smoking and alcohol consumption using logistic regression. Participants of Dutch origin were excluded from the regression analyses, as their PED mean score was close to base value of 1 (i.e., basically no discrimination) with small variation. In the regression analyses, we used two models. In Model 1, we adjusted for ethnicity (in total sample only), age, and sex. In Model 2, we additionally adjusted for marital status, employment status, educational level, and other psychosocial stressors (negative life events and feeling distressed at home). The regression analyses were further presented by ethnicity, as the *p* value for interaction (PED × Ethnicity) was statistically significant for some outcome measures (i.e., *p* value < 0.05) (see Tables 3, 4). All analyses were performed using SPSS version 23.

## Results

In Table 1, the socio-demographic characteristics of the participants are presented. In each group, there were more women than men. Turkish and Moroccan participants were the youngest, were more likely to be married or living with a partner, and were more likely to have no education or elementary education relative to the other groups. Dutch participants had the highest educational attainment and were most often employed, while Turkish and Moroccan participants were most likely not to be in the labour force and Ghanaian participants indicated the highest unemployment rate. Turkish and South-Asian Surinamese participants were more likely to report stress at home. Mean PED scores were around 2.00 for the ethnic minority groups, but the score was somewhat higher for African Surinamese participants (2.05) and lower for Turkish participants (1.84).

**Table 1:** General characteristics of the study sample of the HELIUS study, Amsterdam, The Netherlands, 2011–2015

Variable	Dutch *N* = 4626	South-Asian Surinamese *N* = 3343	African Surinamese *N* = 4414	Ghanaians *N* = 2441	Turkish *N* = 4012	Moroccans *N* = 4290
Sex, male (%)	46.0	46.5	40.5	38.7	44.9	37.9
Age in years, mean (SD)	46.12 (14.05)	45.03 (13.53)	47.55 (12.77)	44.21 (11.53)	39.84 (12.45)	39.70 (13.05)
Marital status (%)						
Never married	32.4	34.3	54.8	35.0	22.6	27.7
Married/living with a partner	57.9	44.1	28.8	36.4	64.0	60.2
Divorced/widowed	9.7	21.6	16.4	28.5	13.5	12.1
Education (%)^a^						
1 (lowest)	3.3	13.9	5.5	27.8	31.2	30.2
2	14.2	33.4	36.2	40.1	25.0	18.2
3	22.0	30.1	36.0	25.9	29.2	34.3
4 (highest)	60.4	22.6	22.3	6.3	14.6	17.3
Employment (%)						
Not in labour force	20.7	23.7	21.2	16.6	32.7	35.7
Unemployed	5.5	15.4	16.4	24.1	14.8	15.8
Employed	73.8	60.9	62.4	59.3	52.6	48.5
*Other psychosocial stressors (%)*						
Stress at home						
Never—some periods	89.0	81.9	86.6	88.7	81.4	82.5
Several periods—constantly	11.0	18.2	13.3	11.3	18.6	17.6
Any life event (in the last 12 months)	59.2	69.8	76.5	58.9	63.3	62.6
Perceived ethnic discrimination						
Mean score, range 1–5 (SD)	1.13 (0.34)	1.97 (0.75)	2.05 (0.77)	1.86 (0.78)	1.84 (0.73)	1.98 (0.79)

^a^1 = no education or elementary education; 2 = lower vocational or lower secondary education; 3 = intermediate vocational or intermediate/higher secondary education, 4 = higher vocational education or university

Table 2 shows the prevalence of smoking and alcohol consumption in different ethnic groups. The highest prevalence of current smoking was seen among participants of Turkish (35.6%), African Surinamese (33.0%), and South-Asian Surinamese (30.6%) origin, while those of Ghanaian origin showed the lowest (4.3%). Moroccan participants have a low prevalence of smoking (13.9%), but among smokers, they have the highest prevalence of heavy smoking (65.9%) and nicotine dependence (39.2%). Prevalence of current drinking was the highest among Dutch participants (91.1%), while the current drinking was the lowest in Turkish (22.3%) and Moroccan participants (7.5%). Alcohol dependence among drinkers was highest among Moroccan participants (32.1%). Similar patterns were observed when using the age- and sex-adjusted odds ratios.

**Table 2:** Prevalence of smoking and alcohol consumption across ethnic groups in the HELIUS study, Amsterdam, The Netherlands, 2011–2015

Outcome variable	Dutch	Ethnicity				
		South-Asian Surinamese	African Surinamese	Ghanaian	Turkish	Moroccan
*Smoking*						
Current smoking						
Prevalence (%)	25.1	30.6	33.0	4.3	35.6	13.9
Odds ratio^a^	1.00	1.31 (1.18–1.45)	1.59 (1.44–1.74)	0.14 (0.11–0.17)	1.59 (1.44–1.75)	0.47 (0.42–0.53)
Heavy smoking/smokers^b^						
Prevalence (%)	49.4	49.0	44.5	35.7	65.5	65.9
Odds ratio	1.00	0.90 (0.75–1.08)	0.71 (0.59–0.84)	0.43 (0.27–0.69)	2.27 (1.91–2.69)	2.13 (1.70–2.67)
Nicotine dependence/smokers^b^						
Prevalence (%)	26.6	39.9	25.4	27.8	40.8	39.2
Odds ratio	1.00	1.79 (1.49–2.15)	0.89 (0.74–1.06)	0.95 (0.59–1.54)	2.09 (1.76–2.49)	1.85 (1.49–2.31)
*Alcohol consumption*						
Current drinking						
Prevalence (%)	91.1	55.4	68.7	47.0	22.3	7.5
Odds ratio^a^	1.00	0.11 (0.10–0.12)	0.22 (0.20–0.25)	0.08 (0.07–0.09)	0.02 (0.02–0.02)	0.01 (0.01–0.01)
Excessive drinking/drinkers^c^						
Prevalence (%)	17.9	6.3	5.9	3.7	4.5	5.1
Odds ratio	1.00	0.29 (0.24–0.36)	0.27 (0.23–0.32)	0.18 (0.13–0.25)	0.22 (0.16–0.31)	0.27 (0.16–0.46)
Alcohol dependence/drinkers^c^						
Prevalence (%)	27.9	15.3	10.2	12.8	18.1	32.1
Odds ratio	1.00	0.39 (0.33–0.45)	0.28 (0.26–0.32)	0.37 (0.31–0.45)	0.39 (0.32–0.47)	0.81 (0.63–1.05)

^a^Adjusted for age and sex, with ethnic Dutch as the reference group

^b^Prevalence of heavy smoking and nicotine dependence within the sample of smokers

^c^Prevalence of excessive drinking and alcohol dependence within the sample of drinkers

Table 3 presents the association of PED with smoking in ethnic minority groups. Total sample analysis shows statistically significant associations between PED and current smoking in model 1 (1.09; 1.04–1.15) and nicotine dependence in model 2 (1.18; 1.09–1.28). No statistically significant associations were found for heavy smoking. In model 2, among African Surinamese participants, 1-unit increase in PED score was significantly associated with 1.16 (95% confidence interval: 1.06–1.27) and 1.34 (1.15–1.57) higher odds of current smoking and nicotine dependence, respectively. Among Ghanaian participants, positive association with heavy smoking was observed (model 2: 2.12; 0.99–4.55). For South-Asian Surinamese participants, the association with nicotine dependence tended to be positive but failed to reach statistical significance. No statistically significant associations with smoking behaviours were observed among participants of Turkish or Moroccan origin.

**Table 3:** Association of perceived ethnic discrimination with smoking behaviours in ethnic minority groups in the HELIUS study, Amsterdam, The Netherlands, 2011–2015

Outcome variable	Total sample^a^	Ethnicity				
		South-Asian Surinamese	African Surinamese	Ghanaian	Turkish	Moroccan
Current smoking						
Model 1^b^	1.09 (1.04–1.15)	1.00 (0.90–1.11)	1.23 (1.13–1.34)	1.03 (0.80–1.33)	1.05 (0.96–1.16)	1.01 (0.90–1.14)
Model 2^c^	1.02 (0.97–1.07)	0.94 (0.85–1.05)	1.16 (1.06–1.27)	0.98 (0.75–1.29)	0.97 (0.88–1.06)	0.93 (0.82–1.05)
Heavy smoking/smokers						
Model 1	1.06 (0.98–1.16)	1.17 (0.99–1.38)	1.09 (0.94–1.25)	2.09 (1.09–4.00)	0.90 (0.77–1.06)	0.99 (0.78–1.27)
Model 2	1.05 (0.97–1.15)	1.17 (0.99–1.39)	1.09 (0.93–1.26)	2.12 (0.99–4.55)	0.89 (0.76–1.05)	0.93 (0.72–1.21)
Nicotine dependence/smokers						
Model 1	1.25 (1.15–1.35)	1.24 (1.05–1.46)	1.42 (1.22–1.64)	1.40 (0.73–2.70)	1.12 (0.97–1.30)	1.21 (0.97–1.51)
Model 2	1.18 (1.09–1.28)	1.19 (1.00–1.42)	1.34 (1.15–1.57)	1.28 (0.57–2.86)	1.06 (0.91–1.16)	1.12 (0.89–1.41)

^a^ *p* values for interaction between perceived ethnic discrimination and ethnicity in the total sample of ethnic minority groups, based on fully adjusted models: current smoking *p* = 0.02, heavy smoking *p* = 0.11, nicotine dependence *p* = 0.25

^b^Model 1 adjusted for ethnicity (in total sample only), age, and sex

^c^Model 2 adjusted for age, sex, marital status, educational level, employment status and other, and psychosocial stressors (negative life events and feeling distressed at home)

The association of PED with alcohol consumption showed a somewhat similar pattern (Table 4). Total sample analyses showed statistically significant associations of PED with current drinking (1.07; 1.02–1.12) and excessive drinking (1.17; 1.03–1.34) in model 1, and with alcohol dependence in model 2 (1.20; 1.09–1.32). In African Surinamese participants, the odds ratio in model 2 for alcohol dependence was 1.40 (1.20–1.64), while the associations with current drinking and excessive drinking tended to be positive but non-significant. In model 2, among Ghanaian participants, 1-unit increase in PED score was associated with 1.21 (1.08–1.36) and 1.26 (0.99–1.60) higher odds for current drinking and alcohol dependence, respectively. In Moroccan participants, we found that PED was negatively associated with current drinking (model 2: 0.68; 0.57–0.81). No associations were observed in South-Asian Surinamese and Turkish participants.

**Table 4:** Association of perceived ethnic discrimination with alcohol consumption in the HELIUS study, Amsterdam, The Netherlands, 2011–2015

Outcome variable	Total sample^a^	Ethnicity				
		South-Asian Surinamese	African Surinamese	Ghanaian	Turkish	Moroccan
Current drinking						
Model 1^b^	1.07 (1.02–1.12)	0.97 (0.88–1.06)	1.08 (0.99–1.18)	1.26 (1.13–1.40)	1.06 (0.95–1.18)	0.82 (0.70–0.95)
Model 2^c^	0.99 (0.94–1.04)	0.94 (0.85–1.04)	1.05 (0.96–1.15)	1.21 (1.08–1.36)	0.92 (0.82–1.04)	0.68 (0.57–0.81)
Excessive drinking/drinkers						
Model 1	1.17 (1.03–1.34)	1.07 (0.84–1.38)	1.30 (1.07–1.57)	1.22 (0.83–1.77)	1.17 (0.75–1.85)	0.94 (0.45–1.96)
Model 2	1.08 (0.94–1.24)	0.96 (0.74–1.25)	1.18 (0.97–1.44)	1.14 (0.77–1.70)	1.08 (0.67–1.74)	0.64 (0.27–1.51)
Alcohol dependence/drinkers						
Model 1	1.32 (1.21–1.45)	1.15 (0.97–1.37)	1.57 (1.35–1.83)	1.32 (1.05–1.65)	1.26 (0.99–1.60)	1.42 (1.02–1.98)
Model 2	1.20 (1.09–1.32)	1.00 (0.83–1.20)	1.40 (1.20–1.64)	1.26 (0.99–1.60)	1.15 (0.90–1.49)	1.21 (0.84–1.75)

^a^ *p* values for interaction between perceived ethnic discrimination and ethnicity in the total sample of ethnic minority groups, based on fully adjusted models: current drinking *p* = 0.002, heavy drinking *p* = 0.50, and alcohol dependence *p* = 0.09

^b^Model 1 adjusted for ethnicity (in total sample only), age, and sex

^c^Model 2 adjusted for marital status, educational level, employment status, and other psychosocial stressors (negative life events and feeling distressed at home)

## Discussion

We found that the associations of PED with smoking and alcohol consumption varied by ethnicity and outcome measure. In general, we observed positive associations in participants of African Surinamese and Ghanaian origin, but no associations in those of South-Asian Surinamese, Turkish or Moroccan origin. Specifically, for smoking, we observed positive associations of PED with current smoking and nicotine dependence in African Surinamese participants and with heavy smoking in Ghanaian participants. For alcohol consumption, the associations were positive for African Surinamese participants for alcohol dependence, and for Ghanaian participants for current drinking.

### Limitations

This study has some limitations. First, the cross-sectional study design does not allow to assess the temporality of the observed associations. Although the previous longitudinal studies have indicated that discrimination precedes poor health outcomes (and not vice versa) (Schulz et al. 2006; Gee and Walsemann 2009), it is unknown whether this also applies to smoking and alcohol consumption. Second, discrimination experiences might not have been measured adequately across the ethnic groups. The EDS (Forman et al. 1997) was developed based on a qualitative study among African American and African Surinamese women (Essed 1991). Lewis et al. (2012) showed that the EDS can be reliably used across different ethnic groups in the US. Third, because smoking and alcohol consumption are generally seen as socially undesirable behaviours in some cultures, there is a chance of underreporting. This might be particularly relevant for Muslims as alcohol is strictly prohibited in Islam (Bush et al. 2003; Dotinga 2005). Finally, findings may have been influenced by confounding bias. Negative personality traits, such as neuroticism, reflect a negative view of oneself and the world (Watson and Clark 1984) and can lead to increased sensitivity to stressful experiences such as discrimination (Bolger and Schilling 2015). This might in turn influence self-reports of PED. Studies have also indicated that people with high neuroticism are more prone to engage in risky behaviours, including smoking and alcohol abuse (Vollrath and Torgersen 2002). However, when we additionally adjusted for neuroticism, the results remained unchanged (see Online Resource 1).

### Interpretation of results

Some ethnic minority groups are shown to have a lower prevalence of smoking and alcohol consumption behaviours compared to Dutch participants, but for Moroccan participants, an interesting pattern emerged. For the whole group, Moroccans smoke and consume alcohol less than Dutch. However, within the group of smokers and drinkers, they exhibit higher rates of heavy smoking and nicotine dependence and similar rate of alcohol dependence relative to Dutch participants. This discrepancy may suggest that Moroccans who smoke or consume alcohol constitute a particular group within the larger community that has a higher susceptibility to more problematic smoking and alcohol behaviours. This higher susceptibility could be hereditary and/or due to higher exposure to social stressors (e.g., disenfranchisement) as this particular group contradicts the cultural and religious values on smoking and drinking within the Moroccan community.

Our study found some positive associations among African-origin groups but no associations in participants of South-Asian Surinamese, Turkish, or Moroccan origin. This is partly in line with literature. The previous literature on the association of discrimination with smoking has largely reported positive associations, while literature on the relationship of discrimination with alcohol consumption found heterogeneous results. The US studies found positive associations between perceived racial/ethnic discrimination and smoking and/or alcohol consumption in African Americans (Bennett et al. 2005; Borrell et al. 2007, 2010), Latinos, Asians and Whites (Landrine et al. 2006; Chae et al. 2008). On the contrary, Yen et al. (1999) found that reaction to unfair treatment did not seem to be associated with heavy drinking, alcohol dependence, or drinks per month among non-white respondents of urban transit operators. Although the underlying reasons for the observed variations in this study remain to be investigated, we suggest three different possible reasons.

First, differential behavioural responses to PED in the ethnic groups might possibly help to understand the ethnic variation. As mentioned, religion and culture play an important role in shaping the smoking and alcohol consumption patterns in some ethnic groups (Kaplan et al. 1990; Dotinga 2005; van Oort et al. 2006). The majority of South-Asian Surinamese, Moroccan, and Turkish participants are Hindu or Muslim, and their religion and cultural values discourage or prohibit smoking and alcohol consumption (Bush et al. 2003; Dotinga 2005; van Oort et al. 2006). Therefore, people of these ethnic minority groups might be less likely to use smoking or alcohol consumption as a means to cope with PED. However, this is not to say that they instead use other (unhealthy) behaviours, such as reduced physical activity or more unhealthy diet. Evidence from the US indicates that perceived chronic discrimination is associated with increased abdominal obesity in a multi-ethnic sample (Hunte and Williams 2009).

Second, the observed variations in associations might be due to differences in how ethnic groups experience ethnic discrimination and/or to differences in the meaning they attach to it. These differences, in turn, may determine health impact. Given the positive associations of PED with smoking and alcohol consumption for both African Surinamese and Ghanaian participants, the impact of discrimination on health behaviours might be related to skin tone, which is an important determinant of discrimination (Perreira and Telles 2014). It could be suggested that those with darker skin tone might experience discrimination in a different way compared to other ethnic groups due to differences in historical background and migration history. People of Turkish or Moroccan origin arrived as temporary guest workers in The Netherlands. In contrast, people of African Surinamese origin have a history of Dutch colonialism, slavery, and racism, which possibly may have shaped their current consciousness and awareness regarding discrimination (Essed 1991).

Third, the ethnic variations in the associations might be related to the differences in the coping resources as used by the ethnic minority groups. It could be possible that people of South-Asian Surinamese, Turkish, and Moroccan origin have more effective coping resources at their disposal to effectively deal with PED. These groups tend to have stronger support networks through family systems and ethnic institutions (Dhami and Skeikh 2000). Studies found that high levels of social support and strong sense of belonging tend to protect against the adverse health effects of discrimination (Kimura 2008).

This study found a negative association between PED and alcohol consumption among Moroccan participants. This negative association might be related to socio-cultural integration, as alcohol consumption might be an indicator of integration into the Dutch society. This implies that Moroccans that experience more discrimination are often less integrated in Dutch society and, therefore, also consume less alcohol. The experience of discrimination in The Netherlands might cause them to adhere to their own religious and cultural values, which discourage alcohol consumption. As such, for Moroccans, more discrimination might be associated with less alcohol consumption.

### Conclusions

Our results indicate that the associations of PED with smoking and alcohol consumption vary across ethnic minority groups. This may suggest that ethnic minority groups differ in how they perceive, cope with, and behaviourally respond to experiences of ethnic discrimination. This should be further studied in in-depth qualitative research. Research should also look into other behavioural responses such as physical activity and dietary intake. This study contributes to the growing European literature on discrimination and health, and also helps to better understand the underlying factors of smoking and drinking patterns in some ethnic minority groups residing in Europe.

## Electronic supplementary material

Below is the link to the electronic supplementary material.

Supplementary material 1 (PDF 181 kb)
