# Supplementary material for: Perceived ethnic discrimination in relation to smoking and alcohol consumption in ethnic minority groups in The Netherlands: the HELIUS study
Source: Int J Public Health. 2017 May 16;62(8):879–87. doi: 10.1007/s00038-017-0977-2 (PMC5641269; doi:10.1007/s00038-017-0977-2)
Supplement: Supplementary file 1 — Supplementary material 1 (PDF 181 kb) [file 38_2017_977_MOESM1_ESM.pdf]

Electronic Supplementary Material

Article title:
Perceived ethnic discrimination in relation to smoking and alcohol consumption in ethnic minority groups in the Netherlands: the HELIUS study

Journal:
International Journal of Public Health

Authors:
Marlies J. Visser[1], Umar Z. Ikram[1*], Eske M. Derks[2,3], Marieke B. Snijder[1], Anton E. Kunst[1].

[1] Department of Public Health, Academic Medical Center, University of Amsterdam, P.O. Box 8 22660, 1100 DD Amsterdam, the Netherlands.
[2] Department of Psychiatry, Academic Medical Center, University of Amsterdam
[3] QIMR Berghofer, Translational Neurogenomics group, Brisbane, Australia

*Corresponding author
E-mail: u.ikram@amc.uva.nl, phone: +31 20 5667441, fax: +31 20 69 72316

Supplementary table 1. The association of perceived ethnic discrimination (PED) with smoking and alcohol consumption in ethnic minority groups, additionally adjusted for neuroticism (besides age, sex, marital status, educational level, employment status and other psychosocial stressors) in the HELIUS study, Amsterdam, the Netherlands, 2011-2015

	Ethnicity				
Outcome Variable	South-Asian Surinamese	African Surinamese	Ghanaian	Turkish	Moroccan
Smoking					
Current smoking	0.94(0.84-1.04)	1.14(1.04-1.25)	0.88(0.67-1.16)	0.94(0.85-1.04)	0.92(0.81-1.04)
Heavy smoking/smokers	1.14(0.95-1.36)	1.06(0.91-1.24)	2.10(0.96-4.59)	0.87(0.74-1.03)	0.92(0.71-1.20)
Nicotine dependence/smokers	1.10(0.92-1.32)	1.29(1.10-1.51)	1.14(0.48-2.67)	0.99(0.85-1.16)	1.08(0.86-1.37)
Alcohol consumption					
Current drinking	0.94(0.85-1.04)	1.06(0.97-1.17)	1.22(1.08-1.36)	0.92(0.81-1.04)	0.65(0.55-0.78)
Excessive drinking/drinkers	0.98(0.75-1.29)	1.11(0.90-1.36)	1.10(0.73-1.66)	1.04(0.63-1.71)	0.60(0.25-1.45)
Alcohol dependence/drinkers	0.93(0.76-1.12)	1.25(1.06-1.48)	1.23(0.96-1.57)	1.08(0.83-1.41)	1.08(0.74-1.58)
